# The Prognosis-Predictive and Immunoregulatory Role of SUMOylation Related Genes: Potential Novel Targets in Prostate Cancer Treatment

**DOI:** 10.3390/ijms241713603

**Published:** 2023-09-02

**Authors:** Jian-Xuan Sun, Ye An, Jia-Cheng Xiang, Jin-Zhou Xu, Jia Hu, Shao-Gang Wang, Qi-Dong Xia

**Affiliations:** Department and Institute of Urology, Tongji Hospital, Tongji Medical College, Huazhong University of Science and Technology, No. 1095 Jiefang Avenue, Wuhan 430030, China; sunjianxuan123@126.com (J.-X.S.); ay121253@163.com (Y.A.); u201910350@hust.edu.cn (J.-C.X.); jason980620@163.com (J.-Z.X.); 2012tj0608@hust.edu.cn (J.H.)

**Keywords:** SUMOylation, prostate cancer, tumor mutation burden, tumor microenvironment, immunotherapy

## Abstract

SUMOylation is an important part of post-translational protein modifications and regulates thousands of proteins in a dynamic manner. The dysregulation of SUMOylation is detected in many cancers. However, the comprehensive role of SUMOylation in prostate cancer (PCa) remains unclear. Using 174 SUMOylation-related genes (SRGs) from the MigDSB database and the transcript data of PCa from The Cancer Genome Atlas (TCGA) and Gene Expression Omnibus (GEO), we constructed a SUMOylation-related risk score and correlated it with prognosis, tumor mutation burden (TMB), tumor microenvironment (TME) infiltration, and response to chemotherapy and immunotherapy. Moreover, we validated two vital SRGs by RT-qPCR, western blotting, and immunohistochemistry. Two vital SRGs (DNMT3B and NUP210) were finally selected. The risk score based on these genes exhibited excellent predictive efficacy in predicting the biochemical recurrence (BCR) of PCa. A nomogram involving the risk score and T stage was established to further explore the clinical value of the risk score. We found the high-score group was correlated with worse prognosis, higher TMB, a more suppressive immune microenvironment, and a better response to Docetaxel but worse to PD-1/CTLA-4 blockade. Meanwhile, we validated the significantly higher expression level of NUP210 in PCa at mRNA and protein levels. This study elucidated the comprehensive role of SUMOylation-related genes in PCa. Importantly, we highlighted the role of an important SRG, NUP210, in PCa, which might be a promising target in PCa treatment. A better understanding of SUMOylation and utilizing the SUMOylation risk score could aid in precision medicine and improve the prognosis of PCa.

## 1. Introduction

Prostate cancer (PCa) is one of the most prevalent malignancies and causes the fifth-highest cancer-related mortality in men worldwide [1]. In 2020, 1.4 million new cases and 375,000 deaths were reported worldwide, and it was estimated to rise to nearly 2.3 million new cases and 740,000 deaths by 2040, attributed to population growth and aging [2]. Radical prostatectomy, radical radiation therapy, and androgen deprivation therapy are common clinical interventions for non-metastatic PCa. Although most patients with low-risk PCa have a favorable prognosis, a subset of patients will still experience disease progression, which may manifest as metastasis or the development of castration-resistant prostate cancer (CRPC). Biochemical recurrence (BCR) is an established clinical phenomenon characterized by an increase in serum prostate-specific antigen (PSA) levels after radical treatment for PCa in the absence of any imaging evidence of disease progression. BCR occurs in a considerable proportion of cases, ranging from 27% to 53% [3]. BCR signals an increased risk of developing CRPC and distant metastases, which demand more intricate treatment strategies and have a poorer prognosis. Consequently, understanding the intrinsic mechanisms and external risk factors associated with BCR is crucial to improving clinical outcomes and mitigating the burden of prostate cancer.

Post-translational protein modifications (PTMs) are essential regulatory mechanisms of cellular proteins that happen during or following translation. The modifications alter the conformation, stability, hydrophobicity, and charge state of proteins and therefore affect their functions in different biological processes [4]. Including ubiquitination, acetylation, phosphorylation, and SUMOylation, more than 450 types of PTMs have been identified to date [5]. SUMOylation is a kind of PTM that depends on the conjugation of small ubiquitin-like modifiers (SUMOs) and targeted proteins. SUMOs are members of the ubiquitin-like family of proteins that weigh about 11 kDa and conjugate with target proteins in the form of single monomers, multiple monomers, or polymers of different types [6]. SUMOs are highly conserved in evolution. Studies indicate that the number of SUMO family members in different organisms is varied, and mammals have five SUMO family members. In mammals, SUMO-5 is a recently discovered SUMO member whose endogenous expression remains to be confirmed, while SUMO-4 probably cannot conjugate to substrates since its maturation is prevented [7]. SUMO-2 is the most abundant member in mammals, and SUMO-2 and SUMO-3 are usually written as SUMO2/3 due to their similar amino acid sequence. As the name suggests, SUMOs are similar to ubiquitin in that they can be modified by other PTMs such as acetylation and/or phosphorylation, and the conjugating process of SUMOs relies on three different types of enzymes: an E1 activating enzyme, an E2 conjugating enzyme, and an E3 ligase. However, it is worth noting that SUMOylation has different functions compared to ubiquitination. The homology of amino acid sequence between SUMO and ubiquitin is only 18%, and the charge distribution of SUMO is quite different from that of ubiquitin [8]. SUMOs are predominantly distributed in the nucleus, where they have a great impact on nucleus processes including gene expression, DNA damage response, RNA reaction, cell cycle progression, nucleocytoplasmic transport, and nuclear formation [6]. SUMOylation plays an important role in regulating different biological processes, and the dysfunction or absence of it contributes to several diseases, such as neuronal disease [9], cardiovascular disease [10], and notably, cancer [11].

Since SUMOylation is crucial for cell survival, the absence of core SUMOylation components in the SUMO machinery cannot lead to a tumor. In fact, dysregulation of SUMOylation has been considered to be related to neoplasms. It has been proven that in many types of cancer, components of SUMOylation were overexpressed, and SUMOylation was significantly upregulated [12]. Previous studies also revealed that ongoing SUMOylation was essential for the progression of the cell cycle in cancer cells [13]. The SUMO E2 conjugating enzyme (also called UBC9) is a widely studied protein in neoplasm and has been found upregulated in many solid tumors, including breast cancer, lung adenocarcinoma, liver cancer, and prostate cancer [14,15,16]. However, interestingly, it was reported that in lung adenocarcinoma and prostate cancer, UBC9 was downregulated in the lymph node metastasis or at the distant metastasis sites [14]. Our previous study demonstrated that ablation of UBC9 in tumor-associated macrophages could reverse the immunosuppressive phenotype of the prostate by promoting macrophage activation and CD8^+^ T cell infiltration, which revealed an inextricable relationship between SUMOylation and tumor microenvironment (TME) [17]. These results indicate that a simple mechanical relationship does not appear to explain the links between SUMOylation and cancer. More comprehensive analyses are needed.

We have constructed a SUMOylation pattern to help explore the SUMOylation characteristics in bladder cancer [18], but the comprehensive role of SUMOylation in prostate cancer still remains poorly understood. Here, we collected 174 SUMOylation-related genes (SRGs) and found the differentially expressed SRGs between tumor tissues and normal prostatic tissues. Using Cox regression and the least absolute shrinkage and selection operator (LASSO) regression, we screened out the differentially expressed SRGs that had prognostic values. Then a SUMOylation risk score was constructed, and patients were stratified into two groups according to the risk score. We correlated the risk score with prognosis, TMB, TME infiltration characteristics, and drug sensitivity. Furthermore, we established a nomogram based on our risk model and T stage to help predict the probability of BCR. Finally, we validated the expression levels of these genes in PCa patients (immunohistochemical analysis) and prostate cancer cell lines (RT-qPCR and western blotting).

## 2. Result

### 2.1. The Identification of Differentially Expressed Prognosis Related SRGs

**Figure 1:**
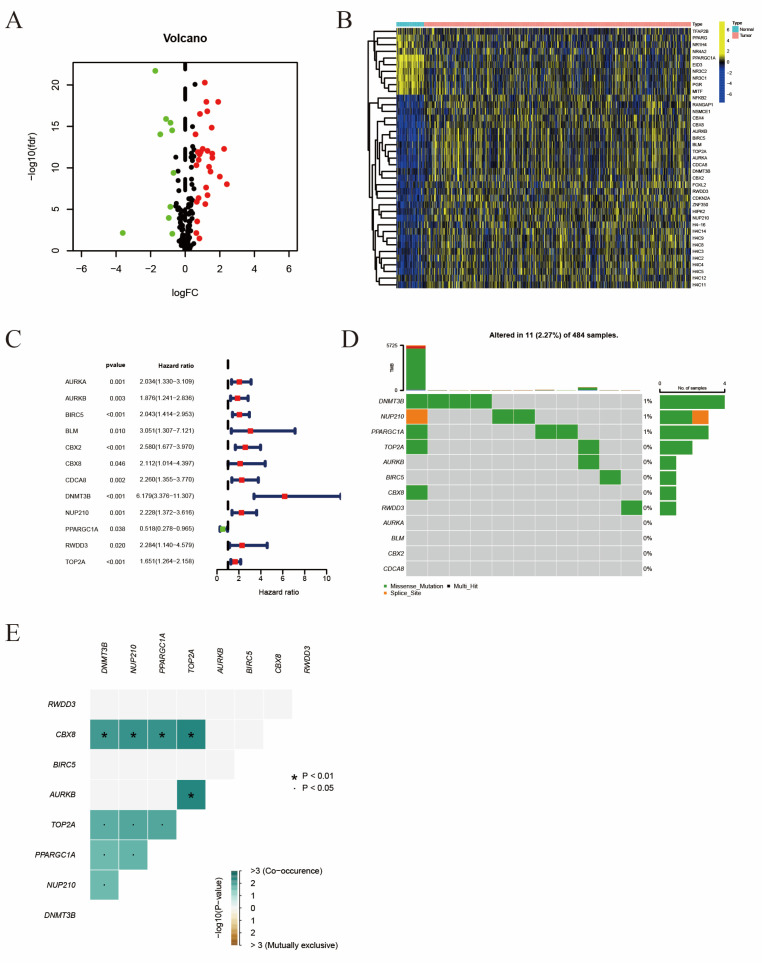
Identification of 12 vital differentially expressed SRGs in PRAD. (**A**) Volcano map of differentially expressed SRGs. (**B**) Heatmap of differentially expressed SRGs. (**C**) Forest plot displayed 12 prognosis-associated genes identified by univariate Cox regression. (**D**) The mutation atlas of these essential SRGs. (**E**) Co-mutation status of these essential SRGs.

We have obtained a total of 663 patients from 3 independent cohorts, including TCGA-PRAD, GSE46602, and GSE70770, and included these patients in the following analyses. The TCGA-PRAD cohort was randomly divided into two cohorts, TCGA_train and TCGA_test, at a 3:2 ratio. The basic clinical characteristics of these patients are summarized in Table 1. Firstly, we extracted SRGs from the MSigDB website and compared their expression levels between prostate cancer tissues and adjacent nontumorous tissues from the TCGA database. The landscape of differentially expressed SRGs was displayed as a volcano plot with a cut-off of |log2FC| > 1 and FDR < 0.05 (Figure 1A) and a heat map (Figure 1B), including 29 up-regulated SRGs and 10 down-regulated SRGs. Then, 11 risky SRGs and 1 protective SRG were finally sorted out using a univariate cox regression model, which was tightly connected to the BCR of prostate cancer (Figure 1C). In addition, the mutation frequency of these 12 genes was only 2.27% in 484 prostate tumor samples, and there were simultaneous mutations among several genes (Figure 1D,E). These findings indicated that these prognostic SRGs were highly stable and connected.

### 2.2. Construction of a Prognostic Model in the TCGA Cohort

**Figure 2:**
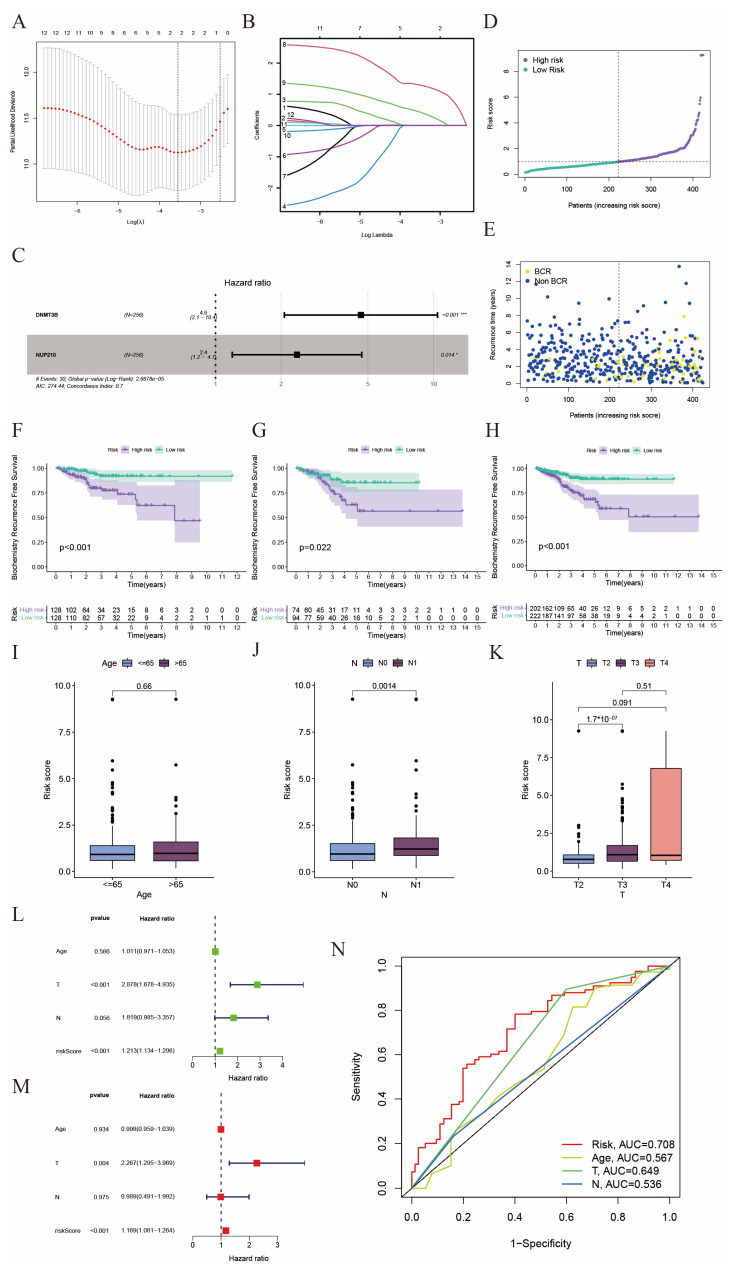
Construction of the prognostic model. (**A**) LASSO coefficient profiles of the expression of 12 candidate genes. (**B**) Selection of the penalty parameter (λ) in the LASSO model via 10-fold cross-validation. The dotted vertical lines are plotted at the optimal values following the minimum criteria (left) and “one standard error” criteria (right). (**C**) Forest plots show the association between selected SRGs and BCR via multivariate Cox regression analysis. (* *p* <0.05, *** *p* <0.001) (**D**) Risk scores of each patient in the TCGA cohort. (**E**) Scatter plot of BCR status of each patient in the TCGA cohort. (**F**–**H**) Kaplan-Meier BCR curves between high- and low-risk groups in the TCGA training cohort, TCGA test cohort, and the whole TCGA cohort, respectively (**I**–**K**) Correlation of clinical features (Age, N staging, and T staging) with risk score. (**L**,**M**) Forest plots show the association between clinicopathological features (including risk score) and prognosis via univariate and multivariate Cox regression analysis. (**N**) ROC curves of prognostic factors.

We used LASSO regression with 10-fold cross-validation to screen out the most important SRGs from the 12 candidate genes (Figure 2A,B). Finally, two SRGs (DNMT3B and NUP210) were selected and used to construct the prognostic model using multivariable Cox regression analysis. The hazard ratios (HR) of these two genes on BCR were shown in Figure 2C, which indicated they were both significantly risky factors in the BCR of prostate cancer. We calculated the risk score for each patient in the TCGA cohort using the formula mentioned above. Then we divided these patients into high-risk (n = 212) and low-risk (n = 212) subgroups with a median value of 1 (Figure 2D). We noticed that the proportion of patients with BCR increased as the risk score grew (Figure 2E), and patients in the high-risk group had worse BCR-free survival than those in the low-risk group in all the TCGA-training cohort (Figure 2F), the TCGA-test cohort (Figure 2G), and the whole TCGA-PRAD cohort (Figure 2H). Compared to all the differentially expressed prognostic SRGs (Figure S1A), the risk score we established could distinguish these patients into two subsets more easily via principal component analysis (PCA) both in the TCGA training cohort (Figure S1B) and the whole TCGA-PRAD cohort (Figure S1C). And the t-Distributed Stochastic Neighbor Embedding (t-SNE) method also indicated that the risk score had good discrimination ability in the TCGA-PRAD cohort (Figure S1D).

Afterwards, we investigated the relationship between risk score and several clinical features, such as age (≤65 and >65) (Figure 2I), N category (N0 and N1) (Figure 2J), T category (T2, T3, and T4) (Figure 2K), M category, and Gleason score (Figure S1E,F). The landscape of the association between the expression levels of two selected SRGs and clinicopathological features (T category, N category, age, and risk score) is shown in Figure S1G. We found a higher risk score was significantly associated with the increasing pathological stage, which implied a worse prognosis. Next, we would like to know whether the risk score could serve as an independent prognostic factor to predict the prognosis of prostate cancer patients. As shown in Figure 2L,M, T category and risk score were independent prognostic predictors for BCR both in univariate and multivariate Cox regression analyses. The area under the curve (AUC) of the ROC curve drawn by the risk score was 0.708, which was higher than age (AUC = 0.567), T category (AUC = 0.649), and N category (AUC = 0.536) (Figure 2N). These results meant the prognostic model we constructed had good specificity and sensitivity.

### 2.3. Validation of the Risk Score in GEO Cohorts

**Figure 3:**
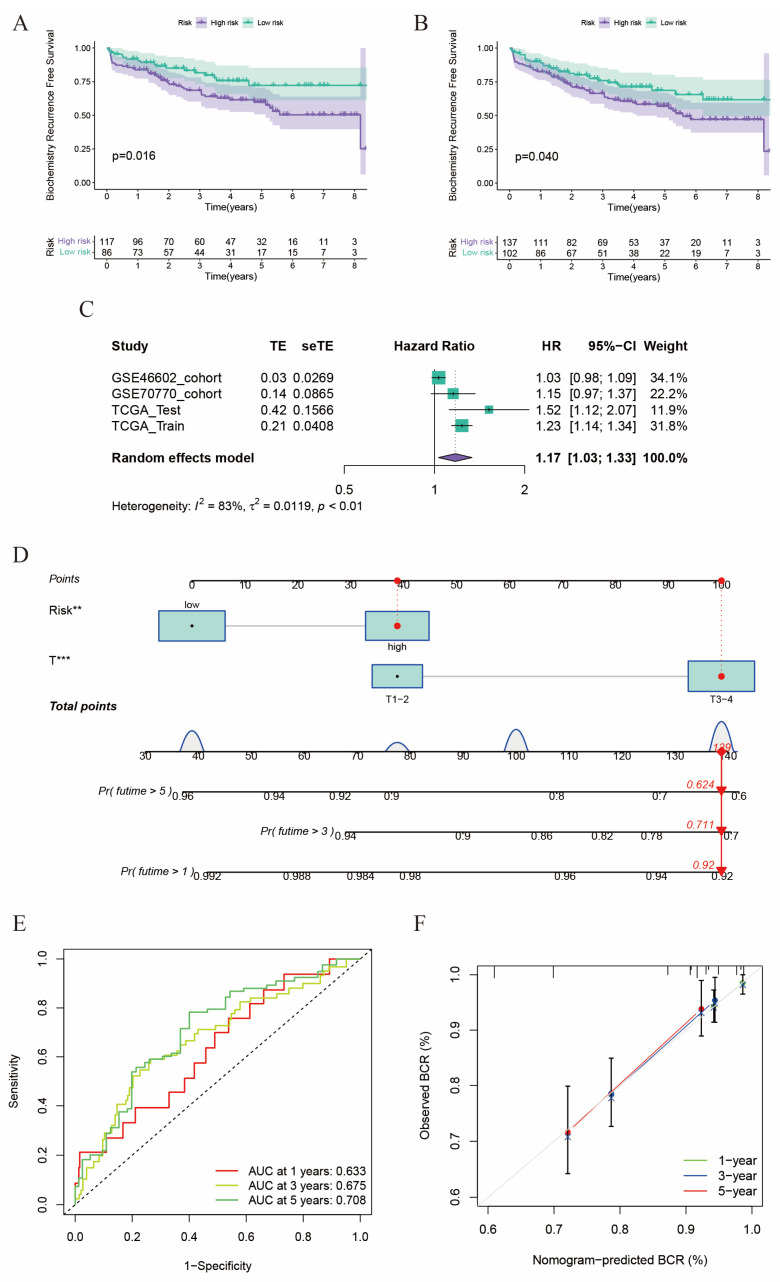
Validation of the risk score in GEO Cohorts. (**A**) Kaplan-Meier curves of patients in two risk groups in GSE70770 cohort. (**B**) Kaplan-Meier curves of patients in two risk groups in meta cohort combined by GSE46602 and GSE70770 cohort. (**C**) Meta-analysis of four cohorts. (**D**) Nomogram was constructed based on risk score and T stage (** *p* < 0.01, *** *p* < 0.001). (**E**) ROC curve of 1-, 3-, and 5-year BCR-free survival. (**F**) Calibration curves of 1-, 3-, and 5-year BCR-free survival.

We have successfully constructed the risk score in the TCGA cohort and verified its tight connection with patients’ prognosis and clinicopathological features. Then, we would like to verify the risk score in two external cohorts in GEO (GSE46602 and GSE70770). We calculated the risk score for each patient in the GSE46602 and GSE70770 cohorts using the formula mentioned above. Then we divided these patients into high-risk and low-risk subgroups with the median value of the risk score (Figure S1H,I). The patients in the GSE46602 and GSE70770 cohorts could be distinguished well by risk score using both PCA and the t-SNE method (Figure S1J–M). Similar to patients in the TCGA cohort, the proportion of patients with BCR was positively connected to risk score in the GSE46602 (Figure S1N) and GSE70770 (Figure S1O) cohorts. Although lacking statistical significance in the GSE46602 cohort (Figure S1P), we noticed that patients in the high-risk group had a significantly worse prognosis, whether in the GSE70770 cohort (Figure 3A) or in the meta cohort combined by the GSE46602 and GSE70770 cohorts (Figure 3B). Then, using a random effects model, we conducted a meta-analysis on the four independent cohorts and calculated the pooled HR of the risk score on the prognosis of prostate cancer (Figure 3C). Unsurprisingly, the pooled HR of risk score was 1.17 (95% CI: 1.03–1.33), which demonstrated that risk score was a valid risky factor for the BCR of prostate cancer.

Subsequently, we constructed a nomogram to predict the probability of BCR in 1, 3, and 5 years for prostate cancer patients using risk score and T stage, which had statistical significance in multivariate Cox regression analysis (Figure 3D). As shown in the nomogram, we randomly selected a patient with a high risk score and T4 stage and calculated the total point of 139, and the corresponding probability of BCR-free survival in 1, 3, and 5 years was 0.92, 0.711, and 0.624, respectively. The AUCs of ROC curves constructed by the nomogram in 1, 3, and 5 years were 0.633, 0.675, and 0.708, which indicated a moderated predictive efficiency (Figure 3E). The calibration curves in 1, 3, and 5 years exhibited excellent consistency in actual BCR probabilities and predicted BCR probabilities (Figure 3F). In summary, the model we established shows robust discrimination ability in external cohorts, and the nomogram worked well in predicting the BCR of the prostate cancer patients.

### 2.4. The Interaction between Tumor Mutation Burden and Risk Score

**Figure 4:**
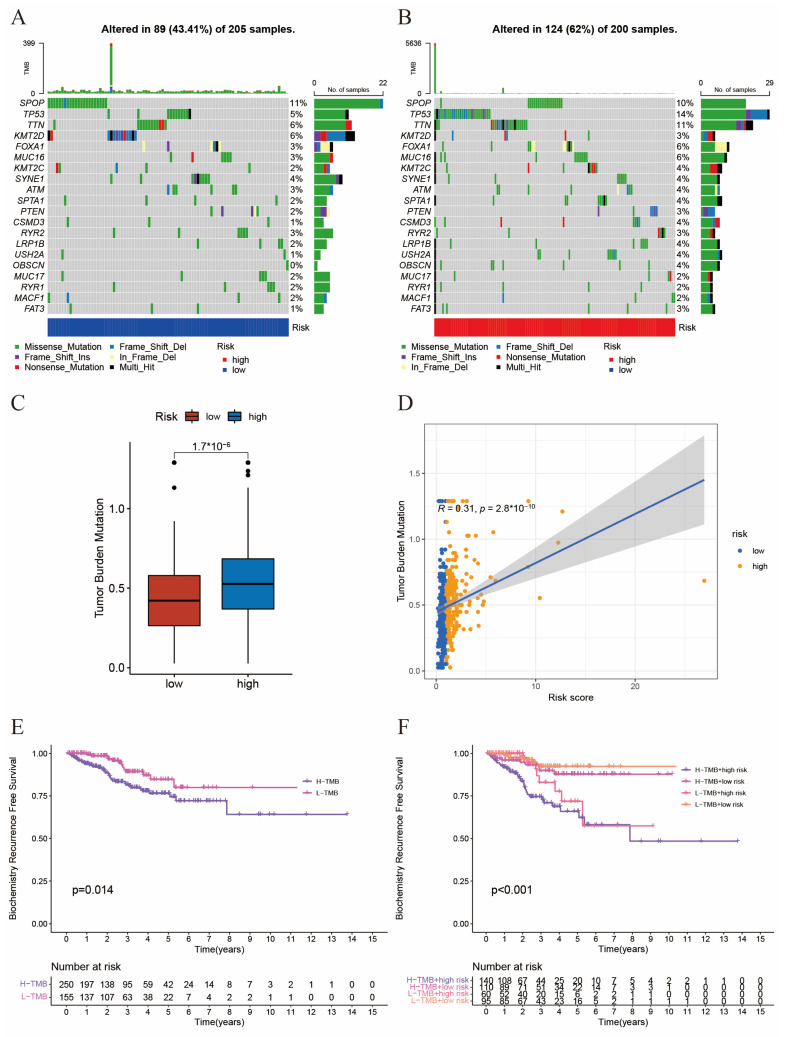
The interaction between tumor mutation burden and risk score. (**A**,**B**) Waterfall plot of tumor somatic mutation established by those with a low risk score (**A**) and a high risk score (**B**). Each column represented individual patients. The upper barplot shows TMB. The number on the right indicated the mutation frequency in each gene. The right barplot shows the proportion of each variant type. (**C**) Comparation of TMB between the two risk groups. (**D**) Correlation of TMB with risk scores. (**E**) BCR-free survival analysis for high- and low-risk patients. (**F**) BCR-free survival analysis for four groups with different patterns with TMB and risk scores.

Next, we would like to explore the interaction between tumor mutation burden (TMB) and risk score. First, we analyzed the frequency of TMB in the top 20 genes in the low-risk (Figure 4A) and high-risk (Figure 4B) groups. We found the total mutation frequency was higher in the high-risk group (62%) than in the low-risk group (43.41%). And for some common mutation loci in prostate cancer, such as TP53 and FOXA1, the mutation frequency was significantly higher in the high-risk group. As shown in Figure 4C,D, TMB was significantly positively correlated with risk score. In addition, patients with high TMB had worse BCR-free survival (Figure 4E). When combining TMB and risk score together, we could divide patients into four subgroups and found patients with a high risk score and a high TMB had the worst clinical outcomes (Figure 4F). Altogether, the combination of TMB and risk score had better predictive capability to predict the prognosis of prostate cancer patients.

### 2.5. The Relationship between Tumor Microenvironment and Risk Score

**Figure 5:**
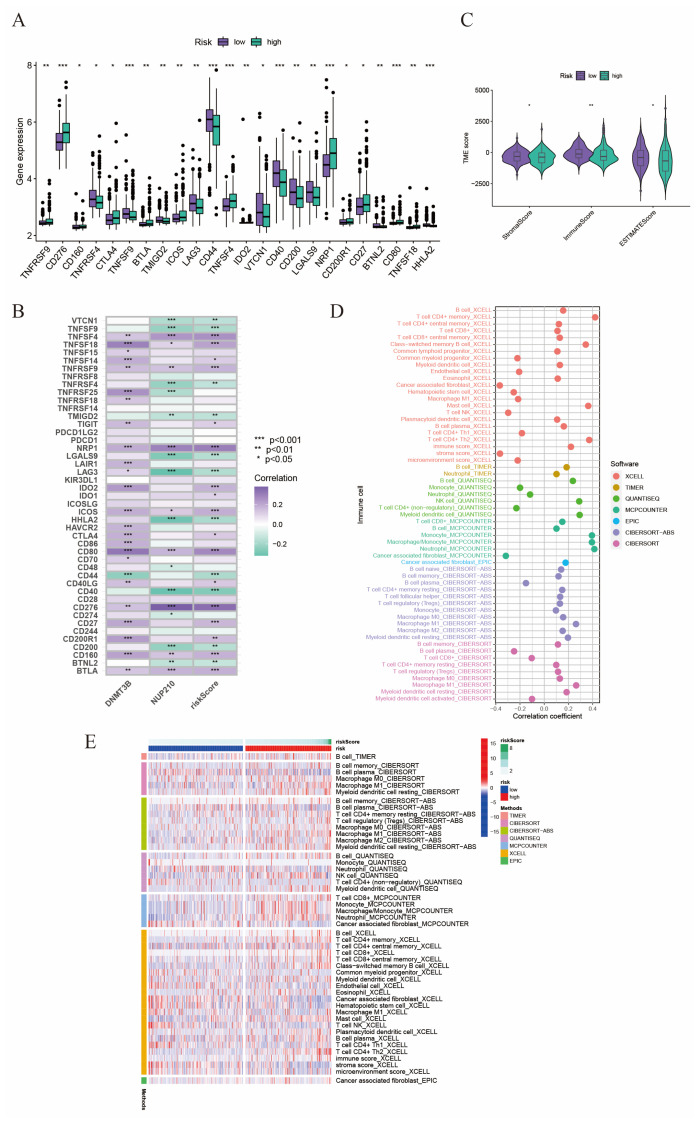
The relationship between tumor microenvironment and risk score. (**A**) The expression level of various immune checkpoints in low- and high-risk subgroups. (**B**) The correlation between immune checkpoints, two SRGs, and risk score. (**C**) Differences in the stromal, immune, and ESTIMATE scores between high- and low-risk groups (Wilcoxon test). (**D**) Correlation of risk scores and immune cell infiltration using seven algorithms. (**E**) The distribution alteration of immune-related cells between the two risk groups. * *p* < 0.05; ** *p* < 0.01; *** *p* < 0.001.

Thereafter, we evaluated the relationship between tumor microenvironment (TME) and risk score. Firstly, we investigated the expression level of various common immune checkpoint (ICP) genes in different risk score groups, and we found most ICPs were upregulated in high-risk groups, such as CD276, CTLA4, and NRP1 (Figure 5A,B). Interestingly, CD276, also known as B7-H3, is a new promising immunotherapy target in various cancers, including prostate cancer. In our study, we found the expression of CD276 was significantly positively correlated with risk score, which indicated that risk score might be a predictor to assess the efficacy of targeting B7-H3 therapy. Additionally, we evaluated the correlation between risk score and three newly discovered markers, ALDH1, CD34, and CD117 (KIT) [19,20], which were reported to be related to PCa progression (Figure S2A–D). We found that the risk score was positively corelated with the expression level of ALDH1. Then, using the ESTIMATE algorithm, we explored the relationship between risk score and tumor purity (Figure 5C). We found the stromal score, immune score, and estimate score were all significantly higher in low-risk groups than those in high-risk groups, which meant a higher tumor purity and lower immune cell infiltration in high-risk groups. Then, we utilized seven algorithms to evaluate the relationship between tumor microenvironment characteristics and risk score (Figure 5D,E). It is worth mentioning that risk score was positively correlated with the infiltration ratio of cancer-associated fibroblasts (Figure S2E), M2 macrophages (Figure S2F), and T-cell regulatory cells (Tregs) (Figure S2G), which are known to lead to immunity suppression and tumor promotion. While risk score was negatively correlated with the infiltration ratio of M1 macrophages (Figure S2H) and CD8^+^ T cells (Figure S2I), which could act as an anti-tumor role.

### 2.6. Drug Sensitivity Analyses in Immunotherapy and Chemotherapy

**Figure 6:**
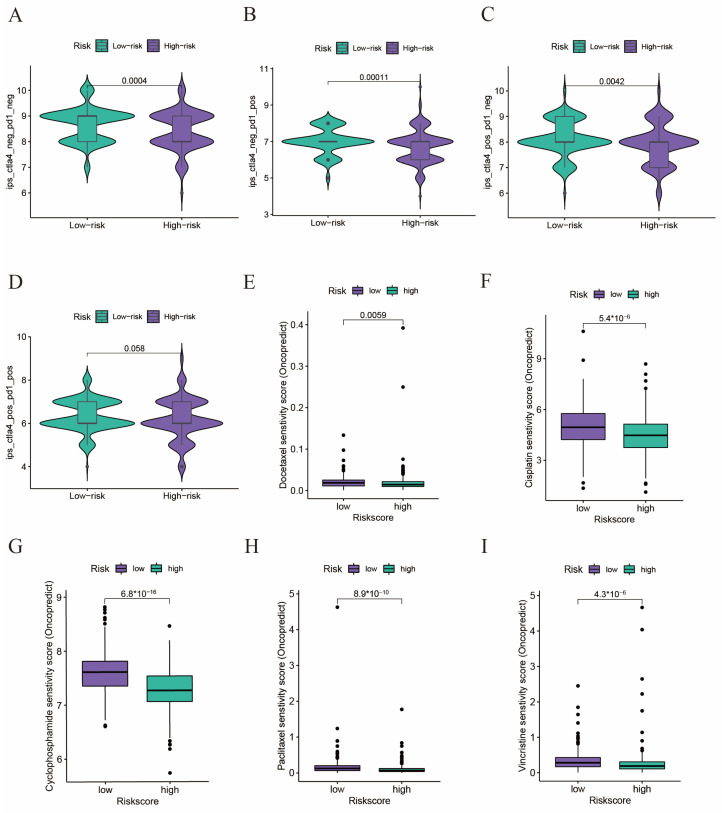
Drug sensitivity analyses in immunotherapy and chemotherapy. (**A**–**D**) The violin diagram shows the differences in response index between high- and low-risk groups among four subgroups. (**E**–**I**) Drug susceptibility analysis. The differences in the response to (**E**) docetaxel, (**F**) cisplatin, (**G**) cyclophosphamide, (**H**) paclitaxel, and (**I**) vincristine between high- and low-risk patients.

We would like to investigate whether the risk score could play a role in predicting the efficacy of immunotherapy and chemotherapy. We used immunophenoscore (IPS) to evaluate the efficacy of immunotherapy, and a higher IPS score meant a better response to immunotherapy. We divided the patients into four subgroups according to their usage of anti-PD-1 and anti-CTLA-4 immunotherapies: CTLA-4 negative PD-1 negative (Figure 6A), CTLA-4 negative PD-1 positive (Figure 6B), CTLA-4 positive PD-1 negative (Figure 6C), and CTLA-4 positive PD-1 positive (Figure 6D). We found the IPS score in all these subgroups was significantly lower than that in the low-risk group, which indicated patients in the low-risk group could benefit more from immunotherapy. Then we used the oncoPredict package to predict the efficacy of some commonly used drugs in chemotherapy, such as docetaxel, cisplatin, cyclophosphamide, cisplatin, paclitaxel, and vincristine, and we found the sensitivity score was significantly lower in the high-risk group, which indicated a better response to chemotherapy (Figure 6E–I). In summary, our results demonstrated that patients with a high risk score might benefit more from chemotherapy than immunotherapy.

### 2.7. Gene Set Enrichment Analysis

**Figure 7:**
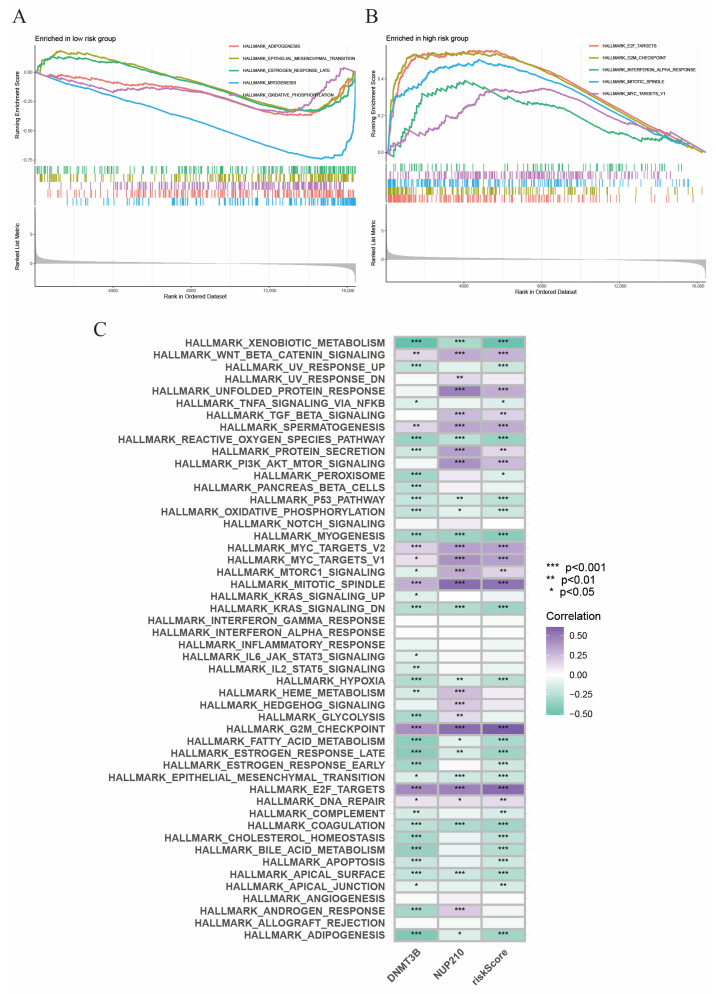
GSEA and GSVA. (**A**) GSEA in low-risk group. (**B**) GSEA in high-risk group. (**C**) GSVA for HALLMARK pathways.

Furthermore, we conducted GSEA to explore the activated signaling pathways in the high- and low-risk subgroups, respectively. We found the top five activated HALLMARK pathways in low-risk subgroups were “adipogenesis”, “epithelial mesenchymal transition”, “estrogen response late”, “myogenesis,” and “oxidative phosphorylation” (Figure 7A). In contrast, the top five enriched signaling pathways in high-risk subgroups were “E2F targets”, “G2M checkpoint”, “interferon alpha response”, “mitotic spindle,” and “MYC targets V1” (Figure 7B). In addition, GSVA showed that risk score was positively correlated with the Wnt/β-Catenin signaling pathway, the PI3K/AKT/mTOR signaling pathway, and the MYC signaling pathway, which were associated with cell growth and tumorigenesis (Figure 7C).

### 2.8. Experimental Verification of Two Vital SRGs

**Figure 8:**
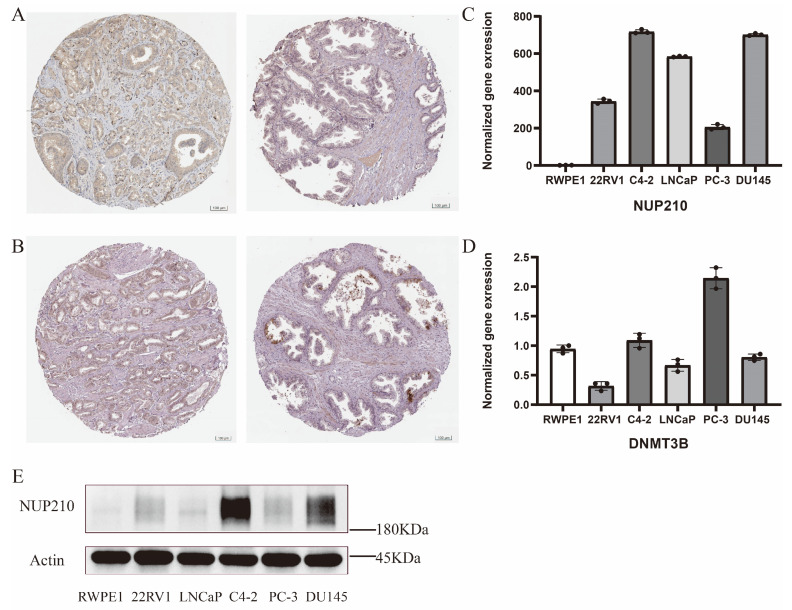
Validation of two vital SRGs in mRNA, protein, and histology levels. (**A**) Immunohistochemical stain for DNMT3B in PCa tissue (left) and normal tissue (right). (**B**) Immunohistochemical stain for NUP210 in PCa tissue (left) and normal tissue (right). (**C**) RT-qPCR reveals the expression level of NUP210 in different cell lines. The expression level of NUP210 in RWPE1 was normalized to 1. (**D**) RT-qPCR reveals the expression level of DNMT3B in different cell lines. (**E**) Western blotting of NUP210. RWPE1 is normal prostatic epithelial cell line, and 22RV1, LNCaP, C4-2, PC-3, and DU145 are PCa cell lines.

Finally, we analyzed the expression of the two vital genes in the SUMOylation risk model. The expression levels of DNMT3B (Figure 8A) and NUP210 (Figure 8B) were upregulated in PCa tissues compared to normal tissues. RT-qPCR reveals expression levels at the mRNA level, and we found that the expression level of NUP210 was significantly higher in PCa cell lines (Figure 8C). Surprisingly, we did not find significant differences in DNMT3B between PCa cell lines and normal prostatic cell lines (Figure 8D), and further research is needed. Western blotting showed that NUP210 was significantly enriched in PCa cell lines rather than normal prostatic epithelial cell lines (Figure 8E).

## 3. Discussion

The initial evidence revealing the association between SUMOylation and cancer derived from the identification of the promyelocytic leukemia protein (PML) and the oncogenic fusion protein PML—retinoic acid receptor-α (RARα) as SUMO substrates [21]. Acute promyelocytic leukemia (APL), a hematological malignancy caused by PML—RARα could be successfully treated by all-trans retinoic acid and trivalent arsenic. Now we know that the degeneration of oncogenic PML—RARα and the restoration of PML are partially ascribed to the hyper-SUMOylation induced by the arsenic component [22,23]. However, this direct correlation between SUMOylation and other cancers does not seem to exist. In fact, when considering the dysregulation of SUMOylation in cancer, not only the SUMOylation cycle-related enzymes but the substrates of SUMO and related functional proteins need to be noted. Research exploring the regulatory role of SUMOylation in biological processes constantly steps forward. Apart from the biological function we mentioned (nucleus-related processes), SUMOylation has been found to play roles in pluripotency, regulation of immunity, and response to oxidative stress [6]. Among them, stress is considered to be the bridge linking SUMOylation and cancer. It has been found that internal or external stress had rapid effects on SUMOylation, and cells were sensitive to stress after the impairment of SUMOylation [24]. ROS, osmotic shock, heat, and ethanol greatly upregulate the SUMOylation in cells via a SUMO2 and SUMO3 manner [25]. SENPs are a family of SUMO proteases that release the SUMOs from substrates. A study elucidated that high concentrations of ROS inactivated SENP and therefore impaired the turnover of SUMOylated substrates [26]. This mechanism may be responsible for the upregulation of SUMOylation when suffering oxidative stress. Given the complexity of tumors and tumor-related environments, cancer cells are more likely to suffer stress such as hypoxia, impaired DNA repair mechanisms, an anti-tumor immune response, and waste removal compared to normal cells. Thus, the upregulated SUMOylation can be viewed as the shelter of cancer cells, stabilizing the complex signaling pathways and helping against intracellular or extracellular stress.

Nowadays, the correlation between SUMOylation and PCa has been studied increasingly, and many SUMOylation-regulated proteins or pathways have been reported to be involved in the development and progression of PCa, such as PTEN, p53, and STAT3 [27,28,29]. In addition, a master factor in PCa, the androgen receptor (AR), has been found to tightly correlate with SUMOylation in the biological process of PCa cells. SUMOylated at two major sites: K520 and K386 by SUMO1 and SUMO2/3, AR acquires decreased transcriptive activity and therefore suppresses PCa [30]. Consistently, mutation at G524 or K386 of AR impairs the SUMOylation and contributes to an enhancement of 2–3 folds of the transcriptive activity of AR in an androgen-dependent manner [30,31]. In this light, the SUMOylation of AR seems to slow the development and progression of PCa. However, it needs to be noted that several cofactors in AR signal pathways, such as FOXA1, AKT, p70, and p68, could reversely regulate the activity of AR after the modifications of SUMO. FOXA1, a pioneer transcription factor, has been found to play important roles in the oncogenesis and development of PCa, which is also known for its high mutation rate in PCa [32]. The impaired SUMOylation of FOXA1 via mutation of SUMO sites (K6, K267, and K389) impairs the proliferation of PCa and LNCaP cells by altering AR nuclear mobility [33]. In general, the SUMOylation regulation in PCa follows a complex regulatory network; there are still many knowns about it. Thus, not only the specific molecules or related pathways, but more comprehensive analyses should be done to further understand the SUMOylation landscape in PCa.

In this article, we first screened out 39 differentially expressed SRGs, of which 29 were upregulated and 10 were downregulated. Just as we mentioned above, the SUMOylation landscape is complex, and not all SRGs are upregulated in PCa patients. Seeler et al. described the SUMOylation in cancer as a “protector” or “enabler”, rather than a “passenger” or “driver” [12]. The typical examples are RAS-driven colorectal cancer and MYC-driven breast cancer, where with insufficient or impaired SUMOylation, cancer cells exhibit specific vulnerabilities differentiating from healthy cells [34,35]. By univariate Cox regression, we screened out 12 SRGs with prognostic values, and only one gene (PPARGC1A) was a protective gene. The mutation atlas revealed general low mutation rates for these 12 genes, which indicated a highly stable feature of SRGs. Since SUMOylation is essential for all cells, including cancer cells, the absence of the core components of the SUMOylation cycle cannot lead to oncogenesis. Only the polymorphisms of UBC9 were found in some cases, and they all led to the poor prognosis for cancers [36,37].

Subsequently, we obtained the two most important SRGs (DNMT3B and NUP210) from 12 candidates by LASSO regression. Interestingly, these two genes are the two most mutated, despite a particularly low mutation rate. DNMT3B, a member of the DNA methyltransferase family, is the de nova methyltransferase, which adds methyl to cytosine bases to establish the DNA methylation model [38]. DNMT3B has been found to be highly correlated with the oncogenesis, growth, metastasis, or prognosis of cancers such as hepatocellular carcinoma, bladder cancer, and papillary thyroid cancer [39,40,41]. Nevertheless, DNMT3B seems to play a opposite role in the oncogenesis of leukemia: high expression of DNMT3B contributes to impaired leukemogenesis, and the absence of DNMTB accelerates the progression of MLL-AF9 [42,43]. There are few studies investigating the relationship between DNMT3B and PCa. Singal et al. reported an increased risk of PCa along with the DNMT3B polymorphisms [44]. Our work highlighted the important role of DNMT3B in PCa, and we hope this could provide some guidance for researchers. NUP210 (Nucleoporin210) is one of the components of nuclear pore complexes (NPCs). NPCs mediate the transport of macromolecules and molecules between the cytoplasm and nucleus, which maintains the division and growth of eukaryotic cells. NPCs are large macromolecule assemblies, and their structures have not been well studied. The AR splice variant-7 (AR–V7) has been considered one of the most important reasons for the development of CRPC. The function of NUP210 in cancer is not clear; however, a study demonstrated that AR–V7 promotes the proliferation of CRPC cells in a NUP210-dependent manner [45]. In addition, NUP210 seems to play a vital role in CD4^+^ T cells; NUP210-deficient CD4^+^ T cells could grow and mature but could not live in the periphery, which exposed the tip of the iceberg of NUP210 [46]. The following construction of a risk score model based on these two genes helps us distinguish patients with high recurrence risk and then guide clinical medication. We found that the risk score was correlated with the clinical features (T stage, N stage, and age). Therefore, for a better understanding of the relationship between risk score and clinical characteristics, we performed COX regression analyses and discovered that risk score and T stage were the independent prognostic factors in PCa patients. A nomogram was constructed to further utilize the risk model.

TMB has always been a focus in cancer research and has been described as a good predictive factor for the therapeutic effectiveness of immune checkpoint blockades (ICBs) in some solid tumors [47]. So, we first investigated the TMB of PCa patients. Generally, we found a higher frequency of somatic frequencies in patients with a high risk score. Some common mutation loci, such as TP53 and FOXA1, were significantly higher in the high-risk group. The tumor-suppressor TP53 is an essential gene in normal cell growth and tumor prevention [48], and the mutation of TP53 frequently indicates a status of more rapid progression, resistance to anti-tumor treatment, and poor prognosis in cancers [49]. Indeed, despite the association between p53 and SUMOylation, along with the crosstalk of other modifications that have been widely studied, the exact role of p53 SUMOylation in oncogenesis still remains unknown [50]. Then, we found that patients in the high-risk group had significantly higher TMB than those in the low-risk group. In the meantime, we found TMB was linearly correlated with risk score, which indicated a possibility that TMB might be a good prognostic predictor.

PCa is a paradigmatic example of a “cold tumor”, which means poor levels of effective immune cell infiltration in TME and a poor response to immunotherapy. Thus, we further investigated the expression levels of ICP genes and immune cell infiltrations in TME. The results demonstrated that the high-risk group was related to a higher level of most ICP genes, which exhibited a potential for ICBs. Notably, we noticed that the expression level of CD276 was significantly positively correlated with risk score and NUP210. These findings demonstrated that the risk score model was expected to be an indicator for guiding the immunotherapy for targeting B7-H3 in PCa [51]. Although immunotherapy is not a commonly used treatment for PCa and several clinical trials ended in failure, pembrolizumab showed promising therapeutic efficacy irrespective of PD-L1 expression level for PCa patients with bone-predominant metastasis (KEYNOTE-199) [52]. In this context, immunotherapy is still a promising direction for the comprehensive management of PCa. Therefore, we next evaluated the response to commonly used chemotherapy drugs for PCa, Docetaxel, and immunotherapy. We found that patients in the high-risk group might benefit more from chemotherapy than immunotherapy. These findings are consistent with the results that patients in the high-risk group usually exhibit higher infiltration levels of M2 macrophages, cancer-associated fibroblasts, and Tregs. These works are of great value for clinical practice because the risk score model is capable of guiding the choice of clinical medications for PCa patients. The GSEA and GSVE analyses showed another SUMOylation landscape. We found classic oncogenesis pathways, including WNT, AKT, and E2F, were highly correlated with SUMOylation risk score and NUP210, which highlighted the role of SUMOylation and NUP210 in PCa. Interestingly, we discovered the unfolded protein response (UPR) was significantly positively correlated with the risk score. We mentioned earlier that due to the rapid proliferation of tumor cells, they faced intercellular and extracellular pressures that blocked growth. UPR here becomes an adaptive mechanism that exploits pro-survival function. UPR is triggered by the accumulation of misfolded or unfolded proteins in the endoplasmic reticulum (ER) and then gives signals and rebuilds ER homoeostasis. Evidence has proved that UPR played a role in the development and progression of glioblastoma multiform, prostate cancer, and breast cancer [53].

At last, we investigated the expression levels of DNMT3B and NUP210 by RT-qPCR, western blotting, and immunohistochemical staining to validate the expression level in mRNA, protein, and histological aspects, respectively. We found that NUP210 showed a significantly higher expression level in PCa than normal tissue in three aspects. However, surprisingly, we did not find a significantly higher expression of DNMT3B in prostate cancer cells via RT-qPCR. This result revealed an inconsistency between results from cell lines and bioinformatics, which suggested that the cancer cell lines could only represent the specific cancer cells from patients to some degree. In general, we found two vital SRGs and constructed a risk model. By utilizing the risk model, we analyzed the prognosis, response to chemo/immunotherapy, and immune infiltration of specific PCa patients. Furthermore, by two factors, we developed a nomogram that shows excellent predictive efficacy in predicting recurrence free survival of PCa. Finally, we confirmed the vital role of NUP210 in mRNA, protein, and histological aspects, which is expected to be a promising target for comprehensive management of PCa.

Nevertheless, there are several limitations to our work. First, since many new SUMOylation-related genes have been discovered, the SRGs used in this work only represent a portion of them and might not be comprehensive enough. Second, the omics data only provided information about mRNA. Although we have validated the expression in cancer cell lines, there is still a lack of external validation from PCa patients in the real world. Third, the number of samples is limited. Finally, the exact mechanisms by which SUMOylation affects development and progression still remain unclear, which might bring bias to our study.

In conclusion, this work elucidated the comprehensive role of SUMOylation-related genes in PCa and gave new insight for the management of PCa. In the meantime, we correlated the risk score with TMB, TME infiltration, prognosis, and response to chemotherapy or immunotherapy. We also established a nomogram model for prognosis prediction for patients with PCa. Importantly, we highlighted the role of NUP210 and provided a new target for further study. A better understanding of SUMOylation and utilizing the SUMOylation risk score could aid in precise treatment and the promotion of the prognosis of PCa.

## 4. Materials and Methods

### 4.1. Cell Culture and the Extraction of RNA and Protein

PCa cell lines (DU145, 22RV1, PC-3, LNCaP, and C4-2) and normal prostatic epithelial cell RWPE1 were purchased from the American Type Culture Collection (ATCC); before all experiments, they were authenticated by short tandem repeat DNA profiling. RWPE1 was cultured in Prostate Epithelial Cell Medium (PEpiCM, ScienCell Research Laboratories 1610 Faraday Ave Carlsbad, CA, USA with 1% penicillin-streptomycin (P/S solution, ScienCell) and Prostate Epithelial Cell Growth Supplement (PEpiCGS, ScienCell). DU145, 22RV1, PC-3, LNCaP, and C4-2 cell lines were cultured in RPMI-1640 with 1% penicillin-streptomycin (Sigma-Aldrich, Saint Louis, MO, USA) and 10% fetal bovine serum (FBS). All cells were incubated at 37 °C with 5% CO_2_. According to the manufacturer’s protocol, we extracted the total RNA or protein using Trizol Reagent (Invitrogen, Carlsbad, CA, USA) or NP40, respectively.

### 4.2. RT-qPCR, Western Blotting and Immunohistochemical Stain

(c) DNAs were obtained by reverse transcription (ReverTra Ace qPCR Kit, Toyobo, Osaka, Japan). Real-time PCR was performed for all genes with primers on a Bio-Rad CFX Connet. Transcript levels were normalized to GAPDH expression and analyzed using the 2^−ΔΔCq^ method. The following PCR primers were designed: human NUP210, F 5′-GGTCATGATCATAGCCTACCACA-3′, R 5′-GCATTGGGAGATGTGGGTGA-3′. Human DNMT3B, F 5′-TGGCAAGTTCTCCGAGGTCTCT-3′, R 5′-GCTGGTCCTCCAATGAGTCTCC-3′. We used the BCA kit (Applygen Technologies, Beijing, China) to obtain the protein concentrations. Protein was separated by SDS–PAGE gel and then transferred to nitrocellulose. The 5% BSA was used to block nitrocellulose membranes, and different antibodies (anti-NUP210, purchased from Abclonal, catalog: A21413; anti-β-actin, purchased from Abclonal, catalog: AC004) were then incubated with nitrocellulose membranes overnight. After incubation of secondary antibodies conjugated with horseradish peroxidase, enhanced chemiluminescence (Thermo Fisher, Shanghai, China) was performed. The immunohistochemical results of two final genes were downloaded from the Human Protein Atlas (HPA) database (https://www.proteinatlas.org/, accessed on 24 June 2023).

### 4.3. Data Sources

The 174 SRGs were obtained from the Molecular Signatures Database (MSigDB, https://www.gsea-msigdb.org/gsea/msigdb, accessed on 4 June 2023). RNA-seq and corresponding clinical information from 663 PCa samples were collected from the Gene Expression Omnibus (GEO) database and The Cancer Genome Atlas (TCGA) database, in which GSE70770 and GSE46602 were used as external validation datasets.

### 4.4. Identification of Differentially Expressed SRGs

At first, the gene expression patterns of each sample were log2 transformed using the package “edgR”. The differentially expressed genes between tumor tissues and normal prostatic tissues were screened out using the R package “limma”, where the filter threshold was set as: |log2 (FC)| > 1 and the false discovery rate was set as: FDR < 0.5. Visualization of gene expression differences was exhibited as a heat map and volcano plot by the R package “pheatmap”. The differentially expressed SRGs were finally identified by an intersection of differentially expressed genes and SRGs. Substantially, we performed univariate Cox regression to select the differentially expressed SRGs which have prognostic values. *p* < 0.05 was set as statistical significance in univariate Cox regression. Considering the essential role of SUMOylation in biological processes, we investigated the mutation atlas and co-mutation status of these essential SRGs in prostate cancer patients.

### 4.5. Construction and Validation of SUMOylation Risk Signature

Having obtained the essential SRGs with prognostic values, we used the least absolute shrinkage and selection operator (LASSO) to complete the final selection of potential genes with nonzero coefficients [54]. The risk signature was constructed based on normalized gene expression levels weighted by corresponding coefficients from the multivariate Cox regression. The SUMOylation score was calculated by the following formula:

$$\mathrm{SUMOylation}\;\mathrm{score}\;=\;\sum_{i=1}^{n}\mathrm{Ki}\;*\;\mathrm{Ei}$$

In this formula, Ei represents the normalized gene expression level of the ith gene, and Ki represents the corresponding coefficient. For better clinical practice, we stratified patients into two groups, namely, the high-risk group and the low-risk group by setting the median risk score as the cut-off value. A survival analysis was performed to evaluate the prognostic differences between these two groups. Apart from the TCGA_PCa cohort, two external cohorts (GSE46602 and GSE70770) were involved to further validate the prediction model. We used the Wilcoxon test to investigate the relevance of the SUMOylation score to clinical features and assessed their independent prognostic values by uni-/multivariate Cox regression. The predictive efficacy was compared among different clinical pathological factors and the SUMOylation risk score by plotting the receiver operating characteristic (ROC) curve. Finally, we constructed a nomogram involving clinical factors and the risk score to predict the probability of BCR.

### 4.6. Tumor Mutation Burden, TME Infiltration, and Drug Response

The tumor mutation burden (TMB) was calculated from the somatic mutation profile of the TCGA_PRAD cohort. The landscape of the top 20 mutant genes from two risk groups was plotted. Then, the correlation between TMB and risk score was evaluated. The infiltration levels of immune cells and somatic cells in TME were estimated by 7 algorithms (QUANTISEQ, CIBERSORT, EPIC, CIBERSORT-ABS, XCELL, TIMER, and MCPCOUNTER) from the TIMER 2.0 database (http://timer.cistrome.org/, accessed on 13 June 2023). Substantially, the correlation between risk score and TME immune cell infiltrations was calculated. In addition, the expression levels of a few important immune checkpoint genes between two different risk groups were compared. The ESTIMATE algorithm was used to investigate the purity of PCa tumors.

To determine whether the risk signature could guide clinical medication, the drug responses to common therapeutic agents were predicted by the R “oncoPredict” package and compared between two risk groups. The Cancer Immunome Atlas (TCIA) was used to assess the response to CTLA-4 or PD-1 blockade of each sample and compared between two risk subgroups [55].

### 4.7. Gene Set Variation Analysis (GSVA) and Gene Set Enrichment Analysis (GSEA)

The HALLMARK pathways were applied to GSEA, which was conducted by the “ClustProfiler” package, and GSVA was performed by the “GSVA” package to determine the correlation between screened candidate genes, risk score, and SUMOylation-related pathways.

## Figures and Tables

**Table 1:** The basic characteristics of included patients.

	Overall	TCGA_Train	TCGA_Test	GSE46602	GSE70770	*p*
n	663	256	168	36	203	
Age (%)						<0.001
41	2 (0.3)	1 (0.4)	0 (0.0)	0 (0.0)	1 (0.5)	
42	1 (0.2)	0 (0.0)	0 (0.0)	0 (0.0)	1 (0.5)	
43	1 (0.2)	1 (0.4)	0 (0.0)	0 (0.0)	0 (0.0)	
44	3 (0.5)	1 (0.4)	1 (0.6)	0 (0.0)	1 (0.5)	
46	6 (0.9)	5 (2.0)	0 (0.0)	1 (2.8)	0 (0.0)	
47	6 (0.9)	4 (1.6)	1 (0.6)	0 (0.0)	1 (0.5)	
48	6 (0.9)	3 (1.2)	1 (0.6)	0 (0.0)	2 (1.0)	
49	6 (0.9)	5 (2.0)	1 (0.6)	0 (0.0)	0 (0.0)	
50	9 (1.4)	4 (1.6)	2 (1.2)	0 (0.0)	3 (1.5)	
51	11 (1.7)	6 (2.3)	3 (1.8)	0 (0.0)	2 (1.0)	
52	11 (1.7)	7 (2.7)	1 (0.6)	1 (2.8)	2 (1.0)	
53	16 (2.4)	8 (3.1)	6 (3.6)	1 (2.8)	1 (0.5)	
54	18 (2.7)	4 (1.6)	9 (5.4)	0 (0.0)	5 (2.5)	
55	25 (3.8)	10 (3.9)	8 (4.8)	1 (2.8)	6 (3.0)	
56	23 (3.5)	9 (3.5)	9 (5.4)	0 (0.0)	5 (2.5)	
57	35 (5.3)	16 (6.2)	11 (6.5)	3 (8.3)	5 (2.5)	
58	26 (3.9)	12 (4.7)	7 (4.2)	3 (8.3)	4 (2.0)	
59	26 (3.9)	9 (3.5)	9 (5.4)	4 (11.1)	4 (2.0)	
60	22 (3.3)	8 (3.1)	8 (4.8)	1 (2.8)	5 (2.5)	
61	32 (4.8)	15 (5.9)	9 (5.4)	1 (2.8)	7 (3.4)	
62	33 (5.0)	13 (5.1)	8 (4.8)	1 (2.8)	11 (5.4)	
63	38 (5.7)	14 (5.5)	11 (6.5)	4 (11.1)	9 (4.4)	
64	32 (4.8)	13 (5.1)	12 (7.1)	1 (2.8)	6 (3.0)	
65	26 (3.9)	13 (5.1)	7 (4.2)	1 (2.8)	5 (2.5)	
66	33 (5.0)	18 (7.0)	11 (6.5)	0 (0.0)	4 (2.0)	
67	29 (4.4)	13 (5.1)	5 (3.0)	3 (8.3)	8 (3.9)	
68	29 (4.4)	14 (5.5)	8 (4.8)	6 (16.7)	1 (0.5)	
69	19 (2.9)	7 (2.7)	4 (2.4)	3 (8.3)	5 (2.5)	
70	12 (1.8)	5 (2.0)	5 (3.0)	0 (0.0)	2 (1.0)	
71	10 (1.5)	6 (2.3)	2 (1.2)	1 (2.8)	1 (0.5)	
72	11 (1.7)	4 (1.6)	5 (3.0)	0 (0.0)	2 (1.0)	
73	6 (0.9)	1 (0.4)	3 (1.8)	0 (0.0)	2 (1.0)	
74	2 (0.3)	1 (0.4)	1 (0.6)	0 (0.0)	0 (0.0)	
75	3 (0.5)	3 (1.2)	0 (0.0)	0 (0.0)	0 (0.0)	
76	1 (0.2)	1 (0.4)	0 (0.0)	0 (0.0)	0 (0.0)	
77	1 (0.2)	1 (0.4)	0 (0.0)	0 (0.0)	0 (0.0)	
78	1 (0.2)	1 (0.4)	0 (0.0)	0 (0.0)	0 (0.0)	
unknow	92 (13.9)	0 (0.0)	0 (0.0)	0 (0.0)	92 (45.3)	
T (%)						0.386
T1–2	253 (38.2)	97 (37.9)	56 (33.3)	19 (52.8)	81 (39.9)	
T3–4	402 (60.6)	157 (61.3)	109 (64.9)	17 (47.2)	119 (58.6)	
unknow	8 (1.2)	2 (0.8)	3 (1.8)	0 (0.0)	3 (1.5)	
BCR_status = BCR/non-BCR (%)	142/521 (21.4/78.6)	30/226 (11.7/88.3)	26/142 (15.5/84.5)	22/14 (61.1/38.9)	64/139 (31.5/68.5)	<0.001
DNMT3B (mean (SD))	2.71 (0.28)	2.69 (0.28)	2.71 (0.27)	2.72 (0.36)	2.72 (0.26)	0.601
NUP210 (mean (SD))	4.88 (0.54)	4.86 (0.55)	4.74 (0.56)	5.07 (0.38)	5.00 (0.48)	<0.001
riskScore (median [IQR])	1.01 [0.69, 1.52]	1.00 [0.62, 1.48]	0.91 [0.58, 1.47]	1.09 [0.83, 1.48]	1.11 [0.81, 1.56]	0.002
Risk = high/low (%)	339/324 (51.1/48.9)	128/128 (50.0/50.0)	74/94 (44.0/56.0)	20/16 (55.6/44.4)	117/86 (57.6/42.4)	0.065

## Data Availability

The datasets presented in this study can be found in online repositories, and the name of them are included in the article or Supplementary Materials.

## Supplementary Materials

The supporting information can be downloaded at: https://www.mdpi.com/article/10.3390/ijms241713603/s1.
