# Supplementary figures and images for: The Prognosis-Predictive and Immunoregulatory Role of SUMOylation Related Genes: Potential Novel Targets in Prostate Cancer Treatment
Source: Int J Mol Sci. 2023 Sep 2;24(17):13603. doi: 10.3390/ijms241713603 (PMC10488061; doi:10.3390/ijms241713603)

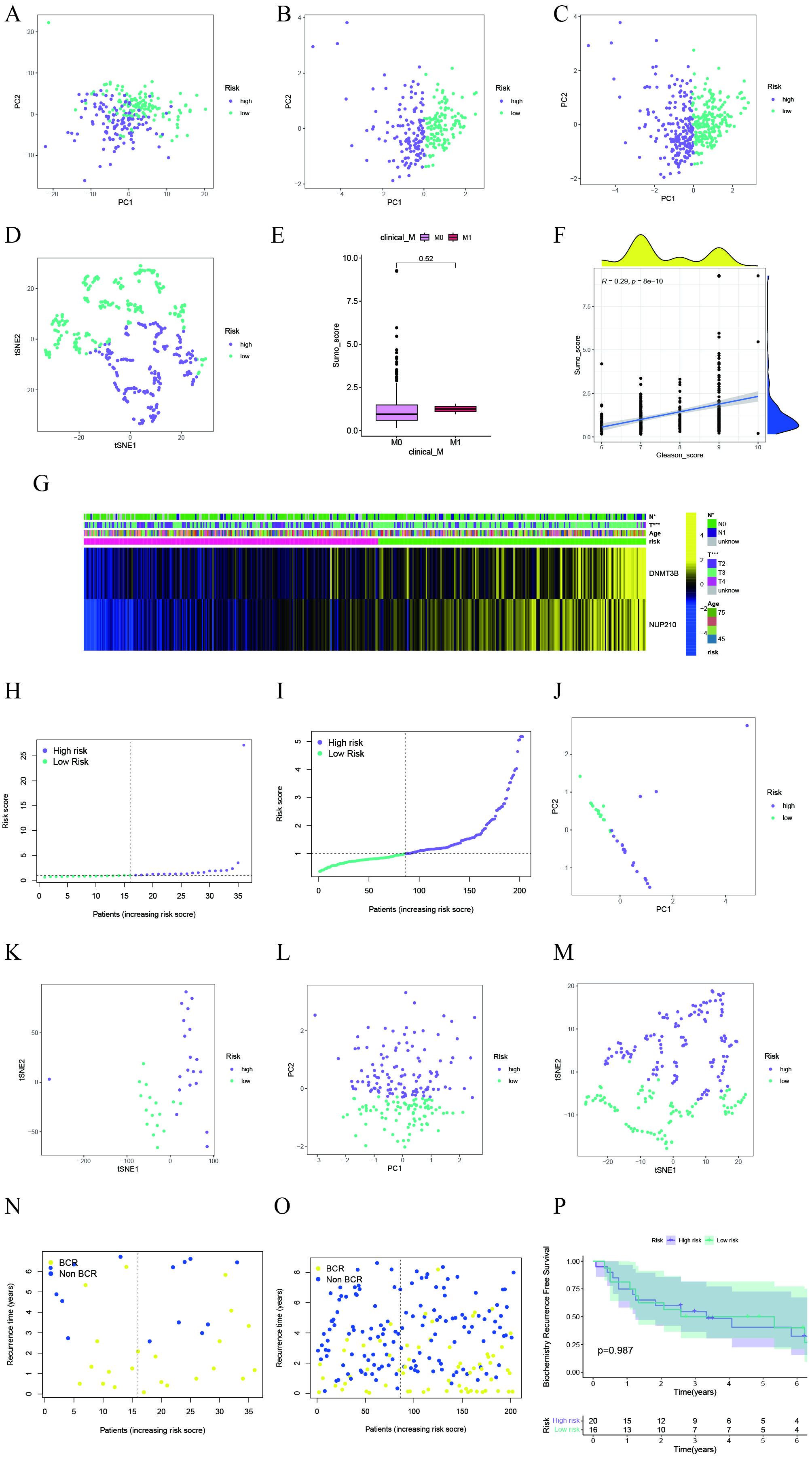

Supplement: Supplementary file 1 [file ijms-24-13603-s001.zip › Supplementary files/Figure S1.jpg]

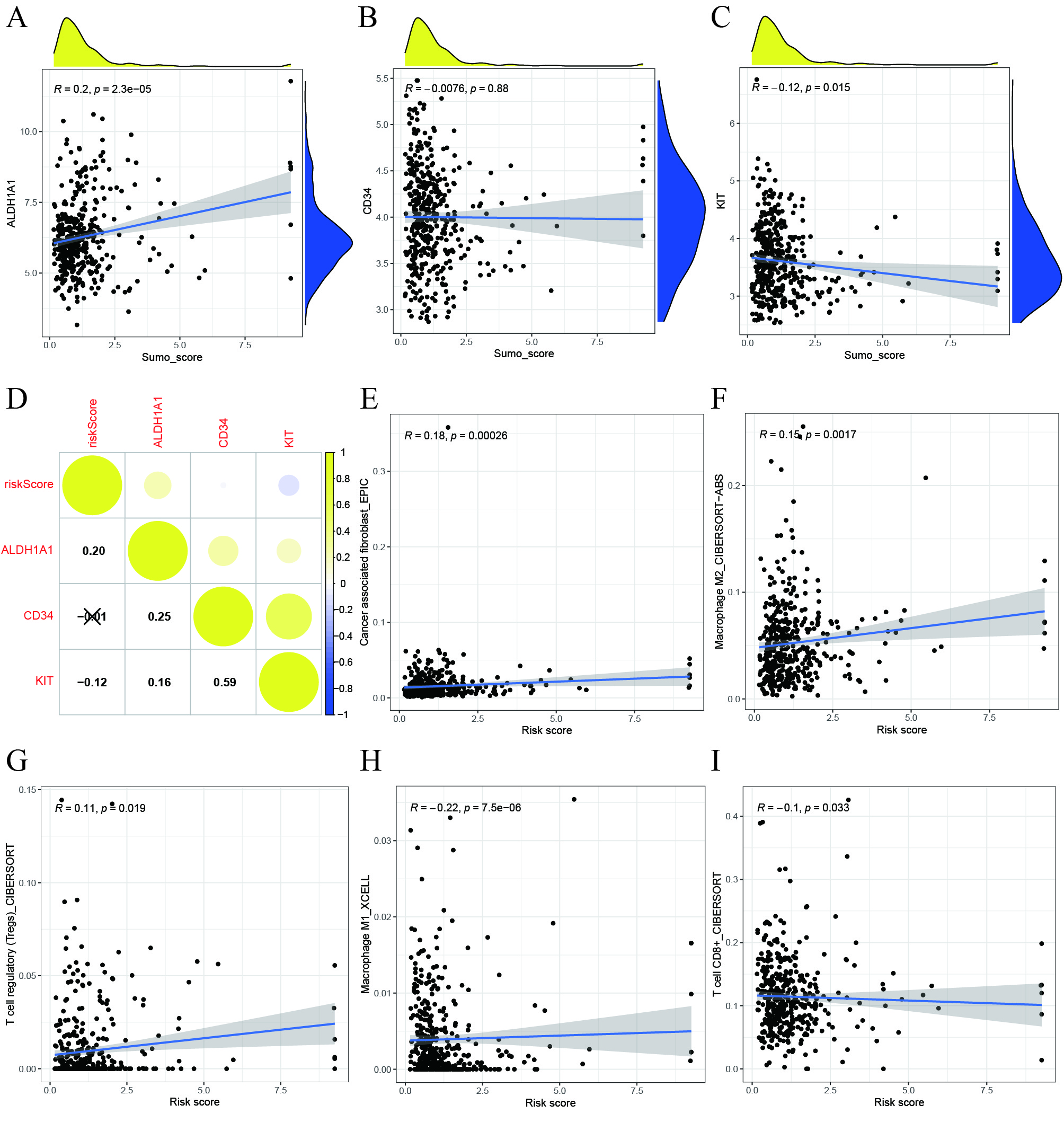

Supplement: Supplementary file 1 [file ijms-24-13603-s001.zip › Supplementary files/Figure S2.jpg]
